# Supplementary material for: Magnetically reshapable 3D multi-electrode arrays of liquid metals for electrophysiological analysis of brain organoids
Source: Nat Commun. 2025 Feb 27;16:2011. doi: 10.1038/s41467-024-55752-3 (PMC11868496; doi:10.1038/s41467-024-55752-3)
Supplement: Supplementary file 1 — Supplementary Information [file 41467_2024_55752_MOESM1_ESM.pdf]

Magnetically reshapable 3D multi-electrode arrays of liquid metals for electrophysiological analysis of brain organoids

Enji Kim [1,2†], Eunseon Jeong [3†], Yeon-Mi Hong[1,2†], Inhea Jeong[1,2], Junghoon Kim [2,3], Yong Won Kwon[1,2], Young-Geun Park[1,2], Jiin Lee[3], Suah Choi[3], Ju-Young Kim[2,4], Jae-Hyun Lee[2,4★], Seung-Woo Cho[2,3,4★], Jang-Ung Park[1,2,4,5,6★].

[1]Department of Materials Science and Engineering, Yonsei University, Seoul 03722, Republic of Korea

[2]Center for Nanomedicine, Institute for Basic Science (IBS), Yonsei University, Seoul, 03722, Republic of Korea

[3]Department of Biotechnology, Yonsei University, Seoul 03722, Republic of Korea

[4]Graduate Program of Nano Biomedical Engineering (NanoBME), Advanced Science Institute, Yonsei University

[5]Department of Neurosurgery, Yonsei University College of Medicine

[6]Yonsei-KIST Convergence Research Institute, Seoul 03722, Republic of Korea

★e-mail: jang-ung@yonsei.ac.kr(J.-U.P), seungwoocho@yonsei.ac.kr(S.-W.C), jhyun_lee@yonsei.ac.kr(J.-H.L)

† These authors contributed equally to this work.

This supplementary information file includes:

Supplementary Figures 1-36

Supplementary Table 1

Supplementary Notes 1-5

Supplementary Figures

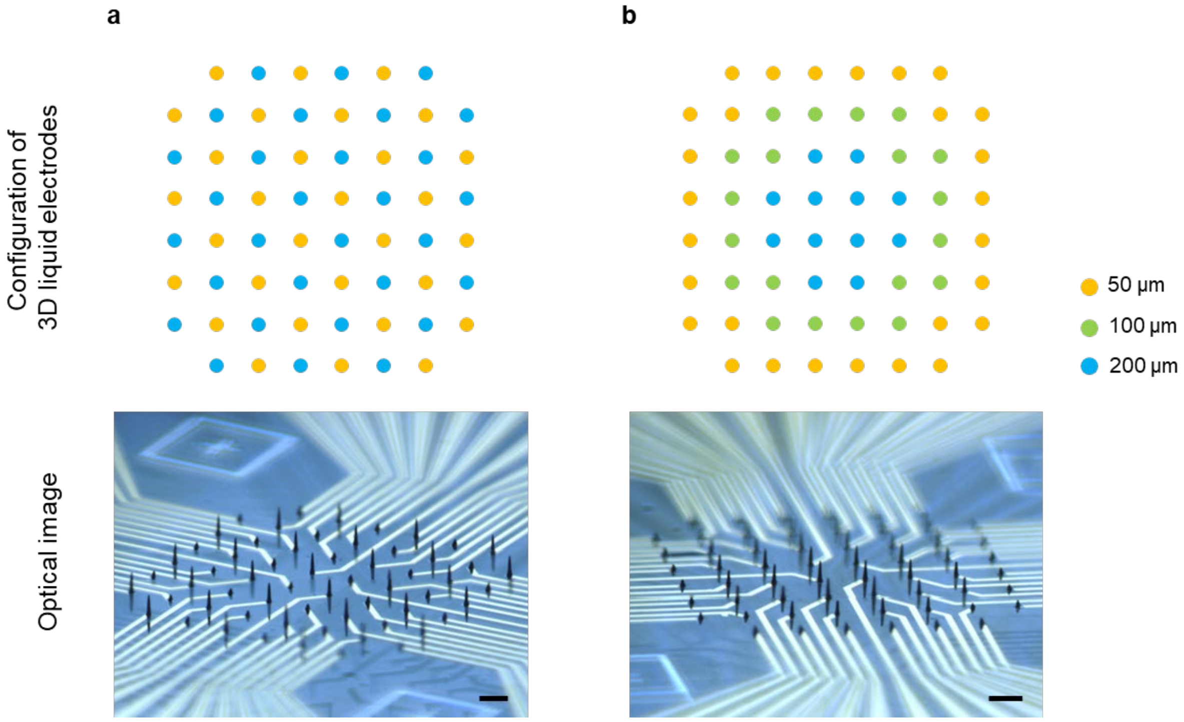

Supplementary Figure 1. Configurations (top) and optical images (bottom) of 3D LM electrodes with various heights. Scale bars, 200 µm.

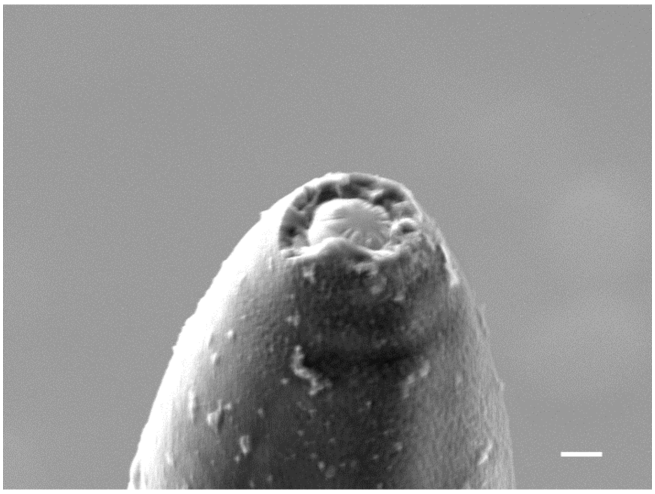

Supplementary Figure 2. Scanning electron microscopy (SEM) image of tip-opened 3D LM electrode. Scale bar, 1 μm. This experiment was independently repeated more than ten times with similar results.

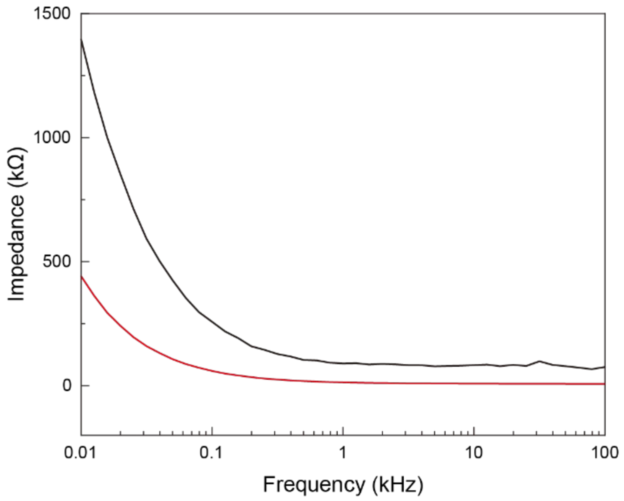

Supplementary Figure 3. Impedance spectroscopy of 3D liquid metal (LM) electrode (black line) and Pt nanoclusters coated LM electrode (red line).

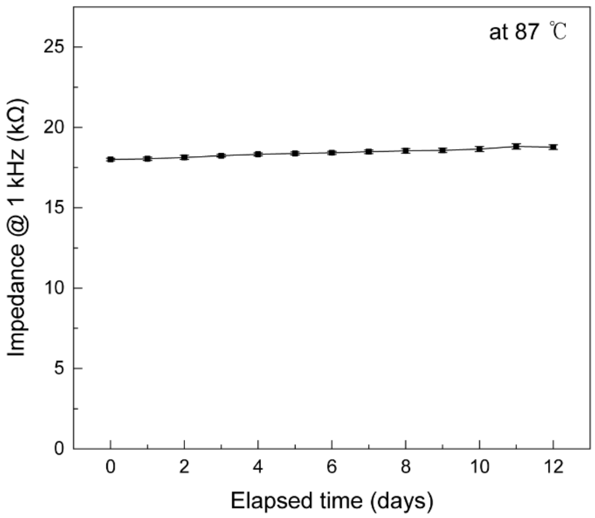

Supplementary Figure 4. Accelerated aging-test of 3D liquid metal (LM) electrodes to investigate the functional stability for long-term neural monitoring of brain organoids. The accelerated aging-test was performed for 12 days at 87 °C, which corresponds to 12 months in the incubator (37 °C). The negligible changes of impedance (18.005 ± 0.083 kΩ to 18.774 ± 0.145 kΩ) indicates the electrical stability of 3D LM electrodes. All data are presented as mean ± s.e.m.

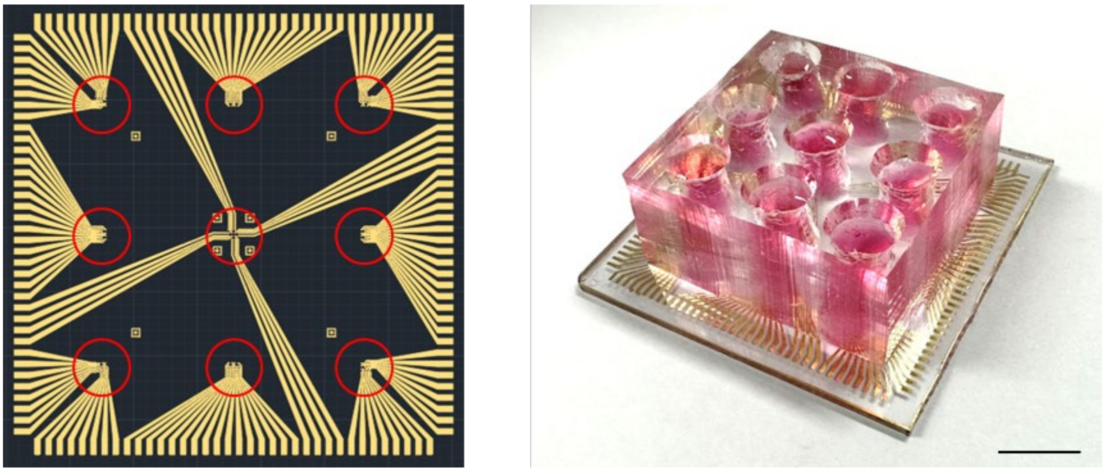

Supplementary Figure 5. Interconnection design (left) and optical image (right) of high-throughput version of 3D LM MEA which can record electrophysiological signals from 9 different organoids simultaneously. The red circles indicate the position of the organoids to be integrated on the device. Scale bar, 1 cm.

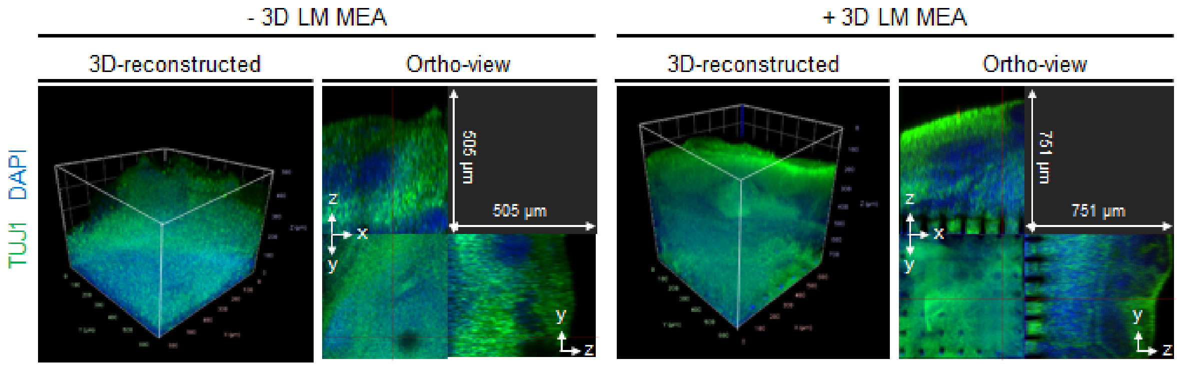

Supplementary Figure 6. Whole-mount 3D imaging of optically cleared organoids without (left) and with (right) inserted 3D LM electrodes. The organoids were stained with TUJ1 and DAPI for investigation of internal architecture of organoids.

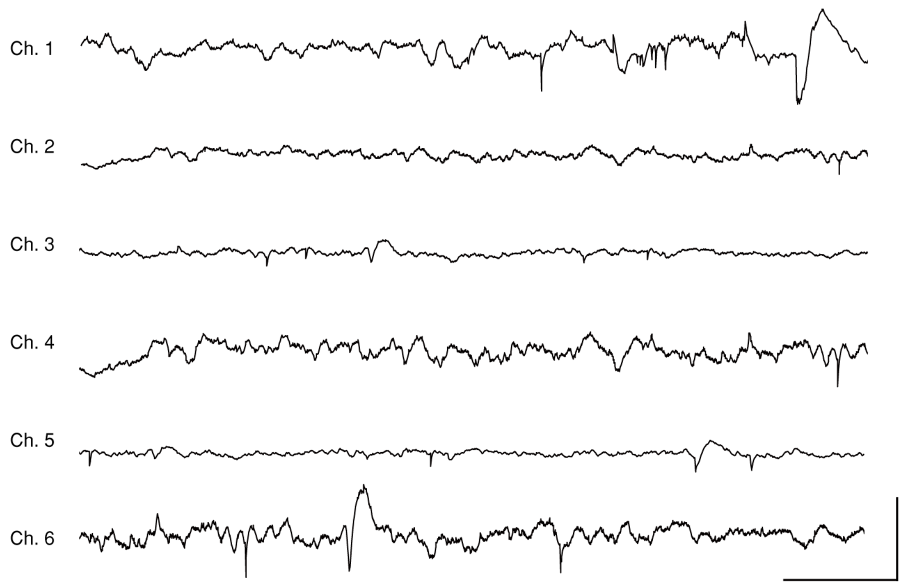

Supplementary Figure 7. Representative local field potentials of a 4-month-old cortical organoid recorded by 3D liquid metal (LM) multi-electrode array (MEA). Scale bars, 500 μV (vertical), 5 s (horizontal).

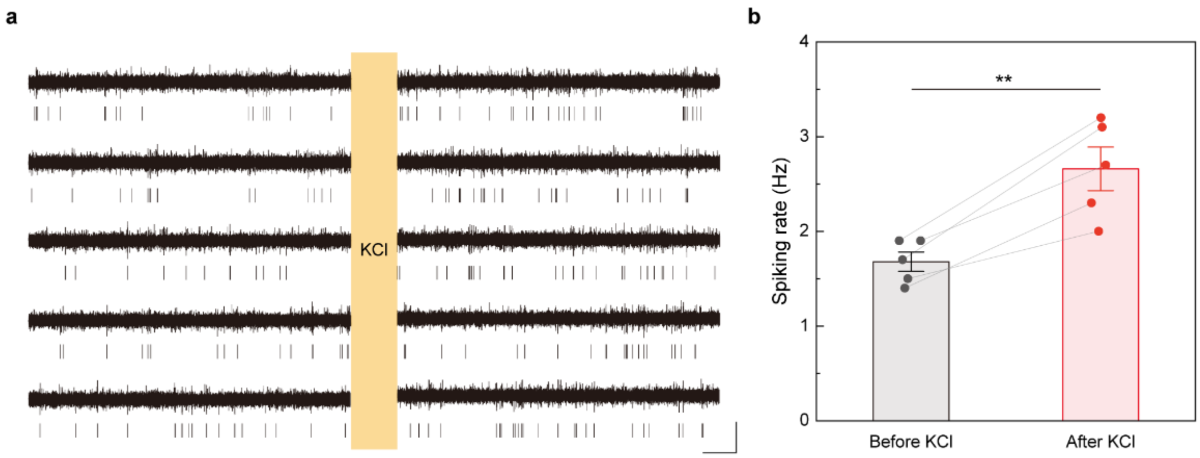

Supplementary Figure 8. Electrophysiological responses of cortical organoids to KCl treatment. **a**, Single unit potentials and raster plots recorded by 5 individual electrodes. Scale bars, 50 µV (vertical), 1 s (horizontal). **b**, Comparison in spiking rates of 5 individual electrodes before and after 50 mM KCl treatment ($p = 0.002258$). All data are presented as mean ± s.e.m., and statistical differences were determined with unpaired, one-sided t-test; $^{**}p<0.01$.

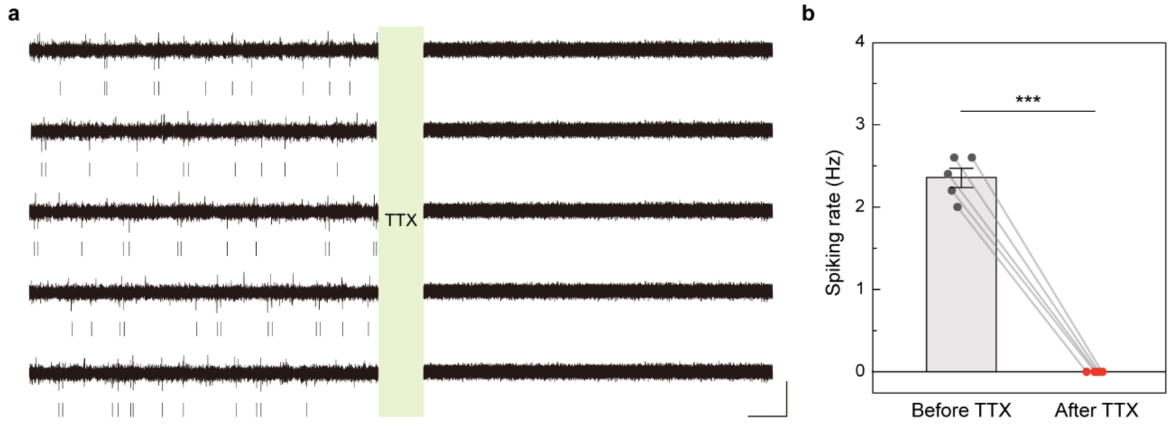

Supplementary Figure 9. Electrophysiological responses of cortical organoids to tetrodotoxin (TTX) treatment. **a**, Single unit potentials and raster plots recorded by 5 individual electrodes. Scale bars, 50 µV (vertical), 0.5 s (horizontal). **b**, Comparison in spiking rates of 5 individual electrodes before and after 1 µM TTX treatment ($p = 1.857\times10^{-8}$). All data are presented as mean ± s.e.m., and statistical differences were determined with unpaired, one-sided t-test; **p<0.001.

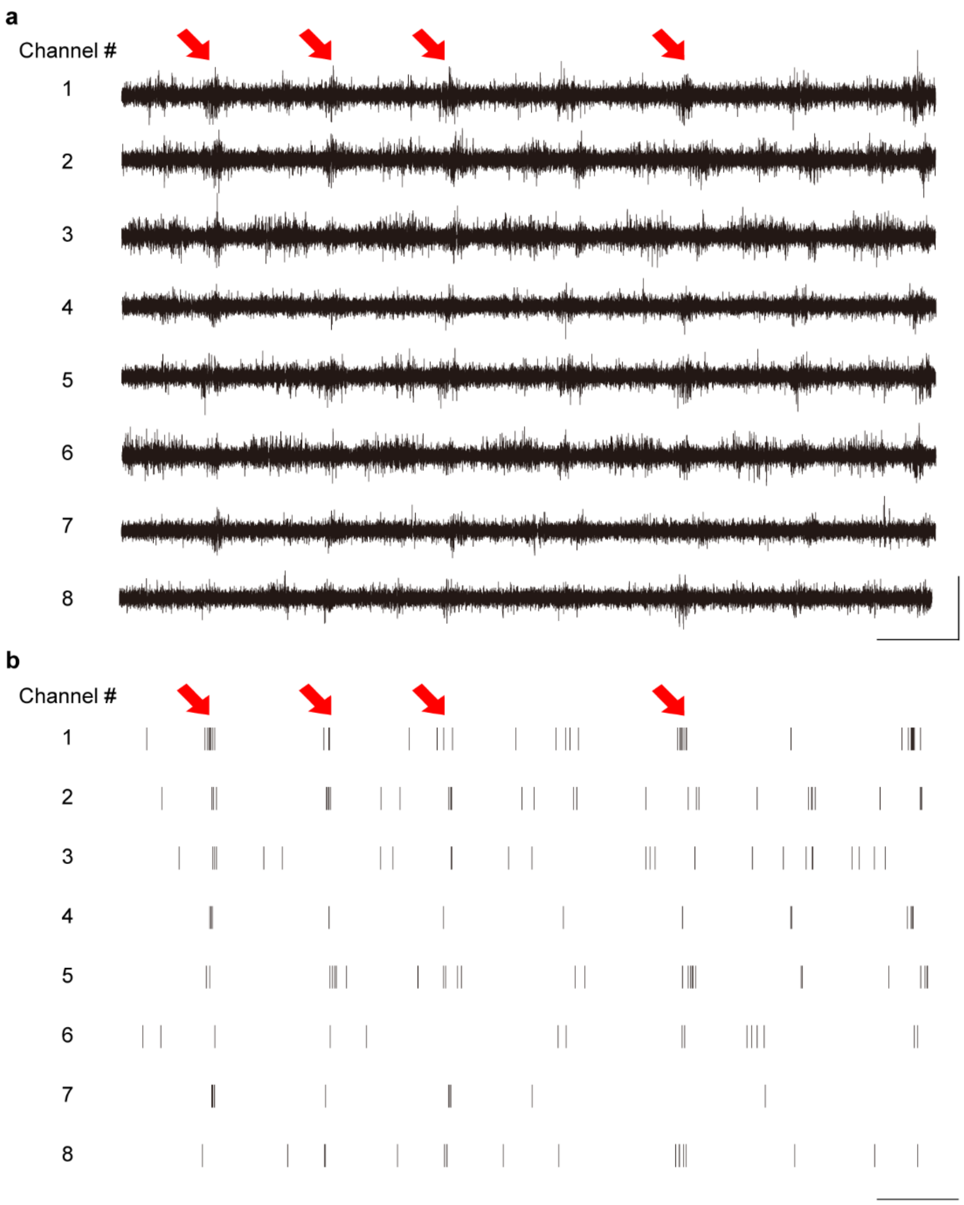

Supplementary Figure 10. Representative synchronized activities of the brain organoid recorded by 3D liquid metal (LM) multi-electrode array (MEA). Red arrows indicate synchronized activities. **a**, Single unit potentials from 8 individual electrodes. Scale bars, 100 μV (vertical), 0.5 s (horizontal). **b**, Raster plots from 8 individual electrodes. Scale bar, 0.5 s.

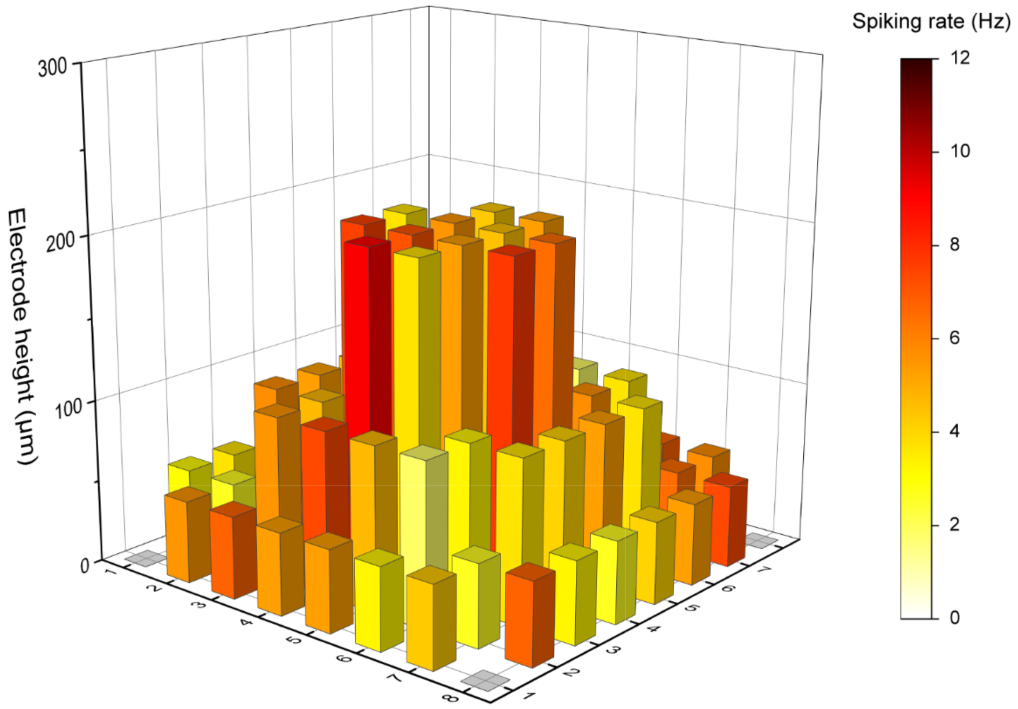

Supplementary Figure 11. Intra-organoid signals recorded by 3D liquid metal (LM) multi-electrode array (MEA) with 3-level of height variance. The spiking rates were observed at various 3D coordinates across the organoid.

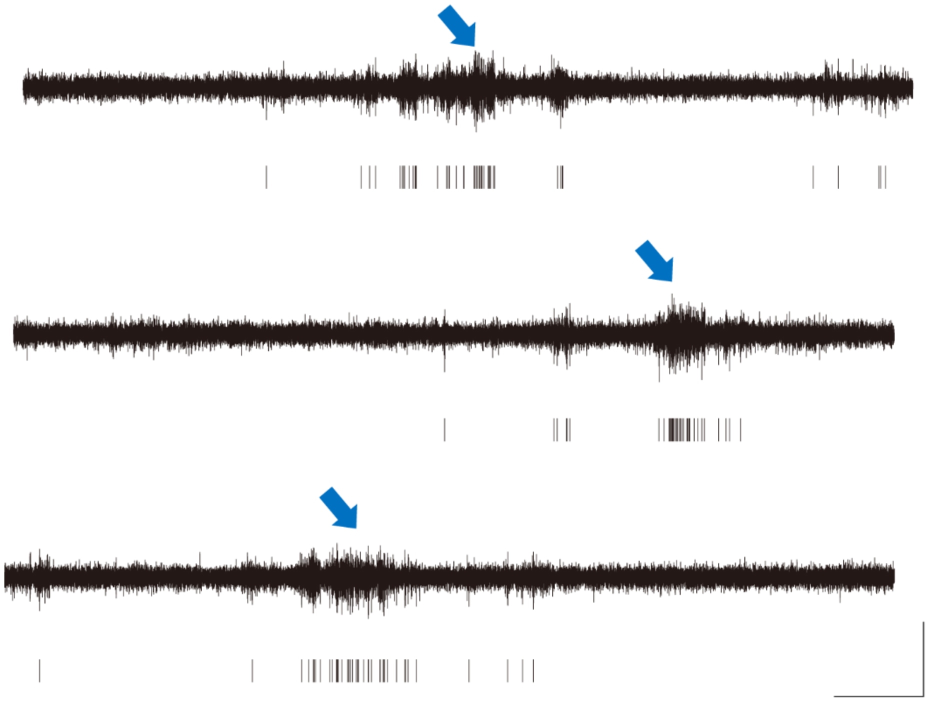

Supplementary Figure 12. Representative bursts of the brain organoid recorded by 3D liquid metal (LM) multi-electrode array (MEA). Blue arrows indicate bursts of neural activities from the organoid. Scale bars, 50 µV (vertical), 0.5 s (horizontal).

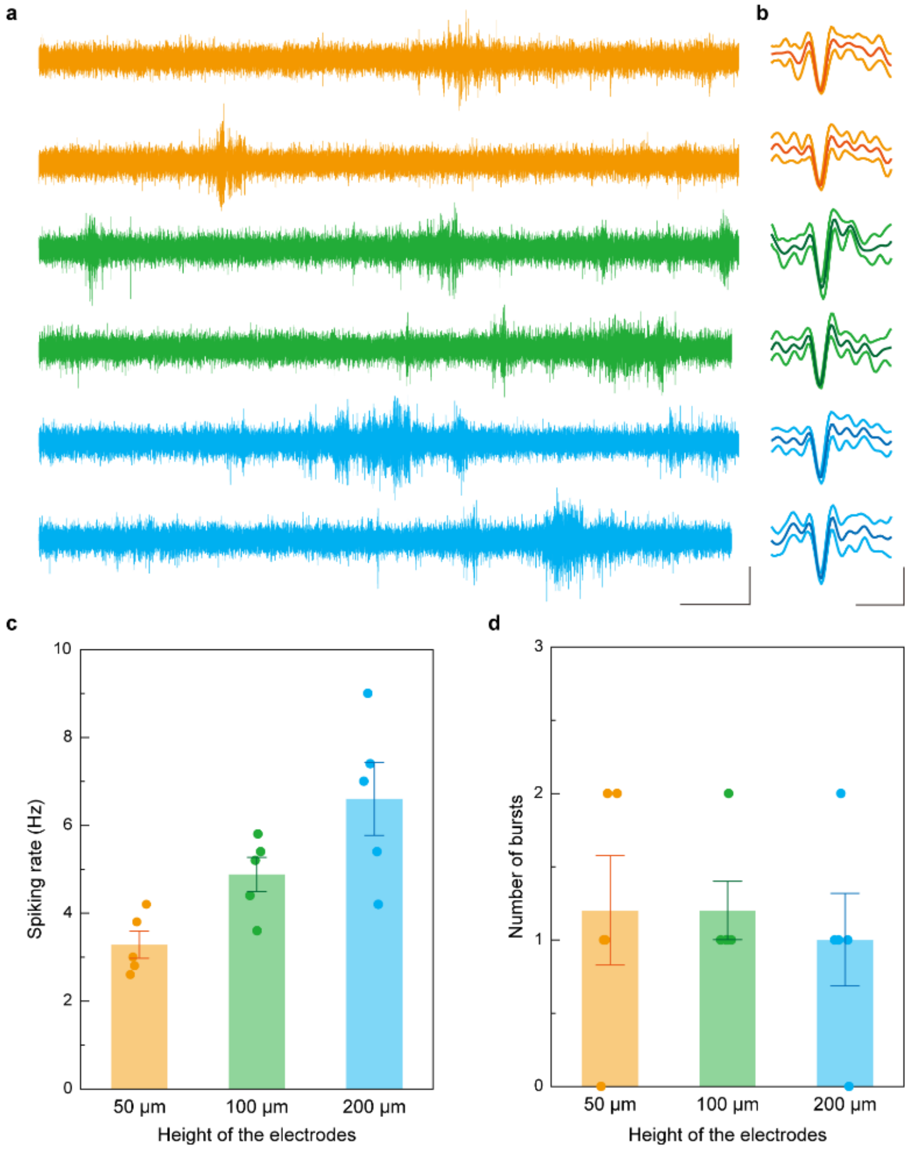

Supplementary Figure 13. Intra-organoid signals recorded by 3D liquid metal (LM) multi-electrode array (MEA) with 3-level of height variance from another organoid. Each color represents the height of the electrodes; Orange, 50 μm, green, 100 μm, and blue, 200 μm. **a,** Representative single-unit potentials depending on the heights of the electrodes. Scale bars, 20 μV (vertical), 0.5 s (horizontal). **b,** Representative spikeforms depending on the heights of the electrodes. Scale bars, 20 μV (vertical), 0.5 ms (horizontal). **c,d,** Changes in neural activities depending on the heights of the electrodes (n = 5 electrodes per each height); (**c**) Spiking rate, (**d**) Number of bursts. All data are presented as mean ± s.e.m.

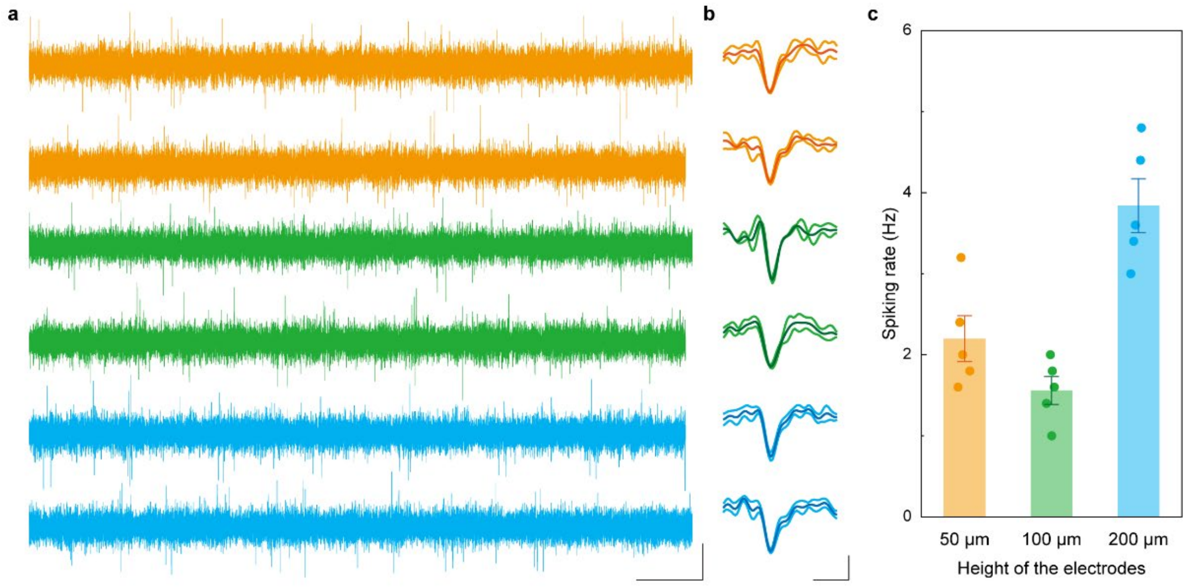

Supplementary Figure 14. Intra-organoid signals recorded by 3D liquid metal (LM) multi-electrode array (MEA) with 3-level of height variance from the other organoid. Each color represents the height of the electrodes; Orange, 50 μm, green, 100 μm, and blue, 200 μm. **a**, Representative single-unit potentials depending on the heights of the electrodes. Scale bars, 20 μV (vertical), 0.5 s (horizontal). **b,** Representative spikeforms depending on the heights of the electrodes. Scale bars, 20 μV (vertical), 0.5 ms (horizontal). **c,** Changes in spiking rates depending on the heights of the electrodes (n = 5 electrodes per each height). All data are presented as mean ± s.e.m.

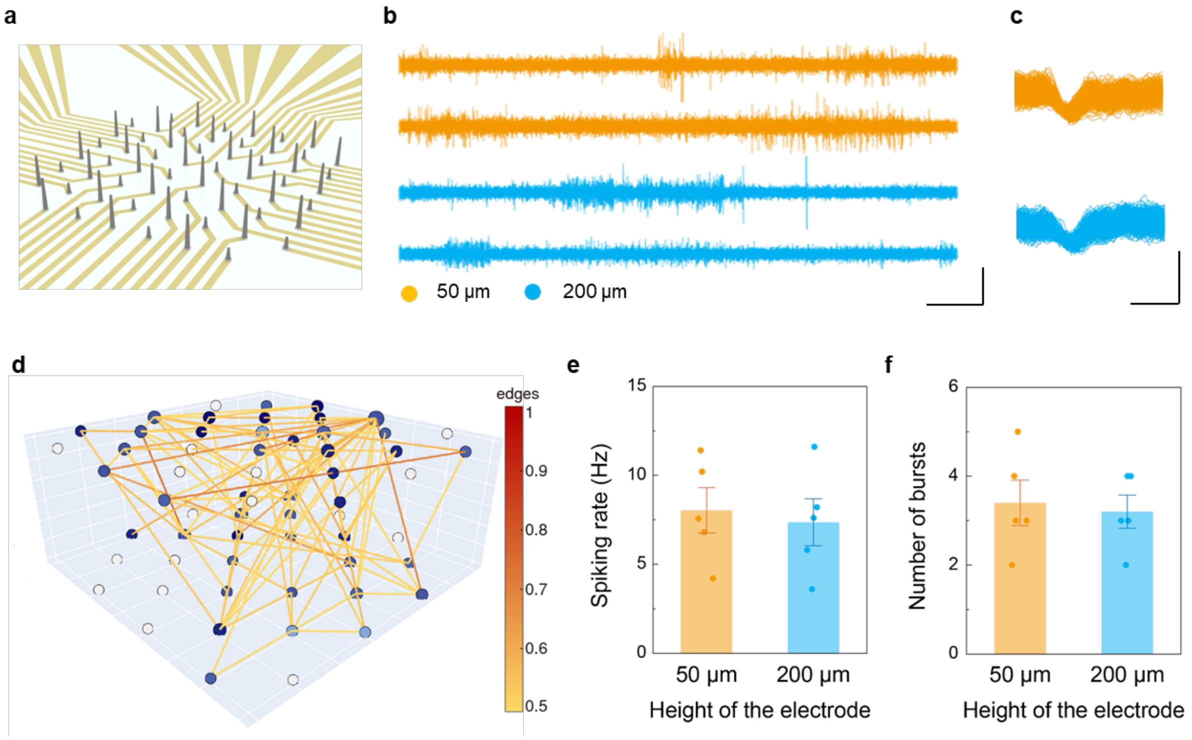

Supplementary Figure 15. Analysis on intra-organoid signals recorded by 3D liquid metal (LM) multi-electrode array (MEA) with 2-level of height variance. **a**, Configuration of 3D LM electrodes with 2-level of heights. **b,c**, Representative intra-organoid signals depending on the heights of the electrodes. Each color represents the height of the electrodes; Orange, 50 µm, blue, 200 µm. (**b**) Single-unit potentials. Scale bars, 100 µV (vertical), 0.5 s (horizontal). (**c**) Spikeforms. Scale bars, 100 µV (vertical), 0.5 ms (horizontal). **d**, 3D neural networking map corresponding to the 2-level height variance of 3D LM MEA. **e,f**, Changes in neural activity depending on the heights of the electrodes (n = 5 electrodes per each height); (**e**) Spiking rate, (**f**) Number of bursts. All data are presented as mean ± s.e.m.

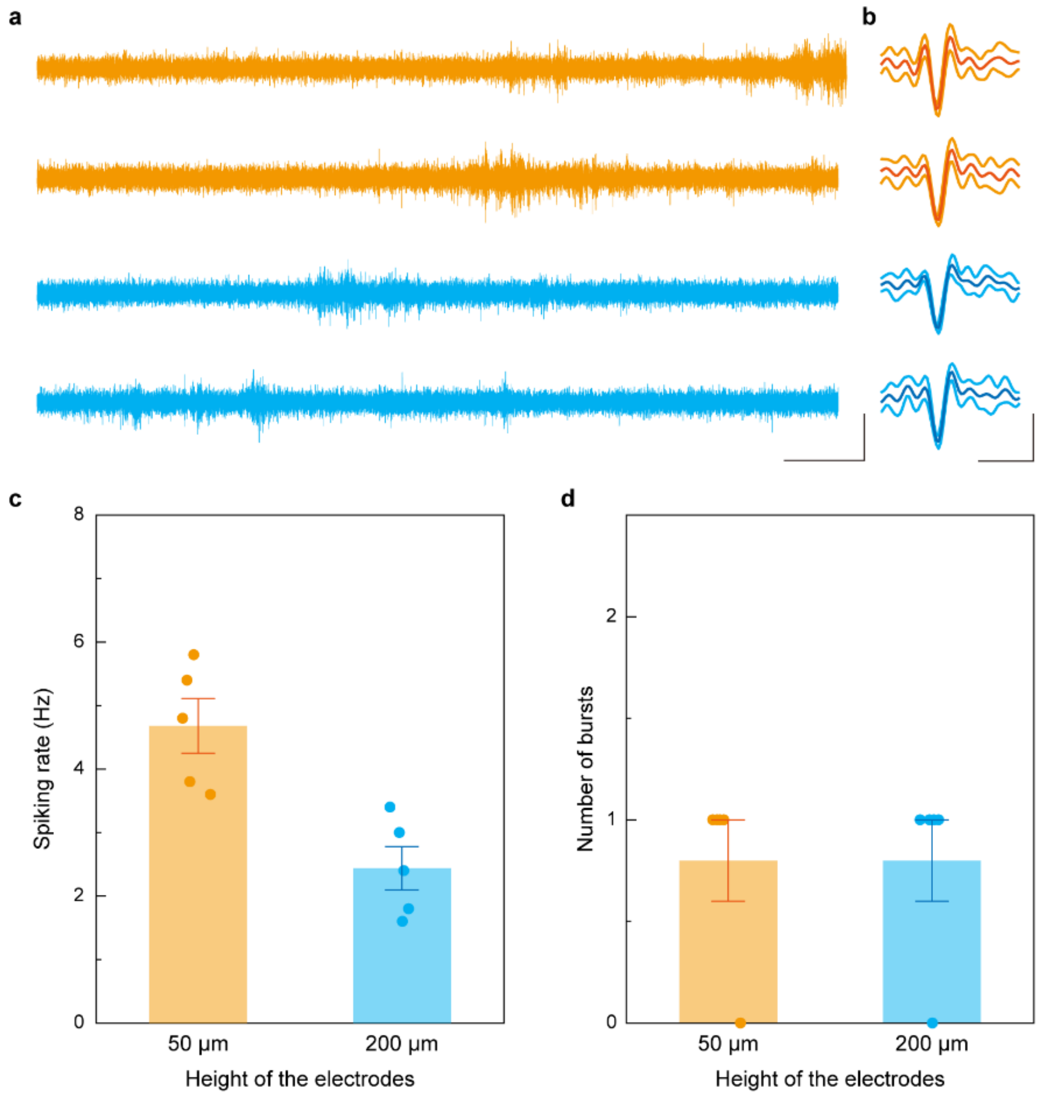

Supplementary Figure 16. Intra-organoid signals recorded by 3D liquid metal (LM) multi-electrode array (MEA) with 2-level of height variance from another organoid. Each color represents the height of the electrodes; Orange, 50 μm, and blue, 200 μm. **a**, Representative single-unit potentials depending on the heights of the electrodes. Scale bars, 50 μV (vertical), 0.5 s (horizontal). **b,** Representative spikeforms depending on the heights of the electrodes. Scale bars, 50 μV (vertical), 1 ms (horizontal). **c**,**d,** Changes in neural activities depending on the heights of the electrodes (n = 5 electrodes per each height); (**c**) Spiking rate, (**d**) Number of bursts. All data are presented as mean ± s.e.m.

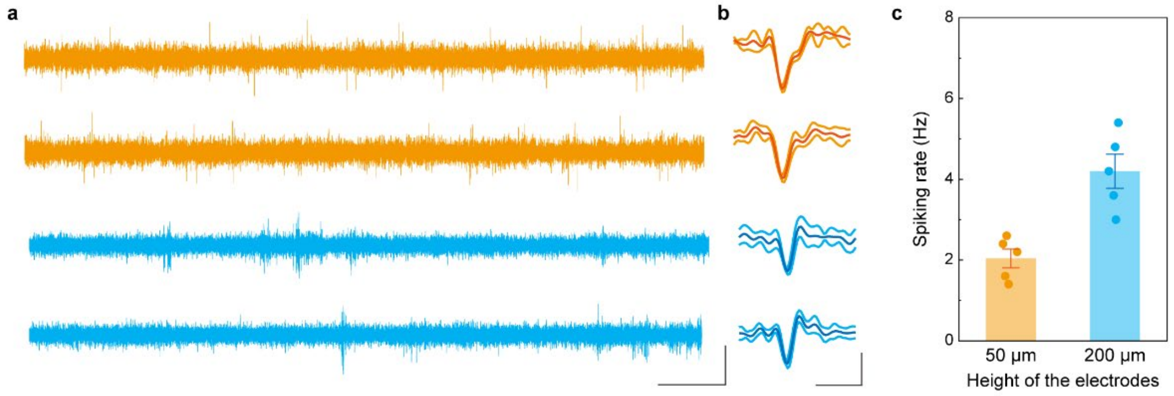

Supplementary Figure 17. Intra-organoid signals recorded by liquid metal (LM) multi-electrode array (MEA) with 2-level of height variance from the other organoid. Each color represents the height of the electrodes; Orange, 50 μm, and blue, 200 μm. **a**, Representative single-unit potentials depending on the heights of the electrodes. Scale bars, 50 μV (vertical), 0.5 s (horizontal). **b,** Representative spikeforms depending on the heights of the electrodes. Scale bars, 50 μV (vertical), 1 ms (horizontal). **c**, Changes in spiking rates depending on the heights of the electrodes (n = 5 electrodes per each height). All data are presented as mean ± s.e.m.

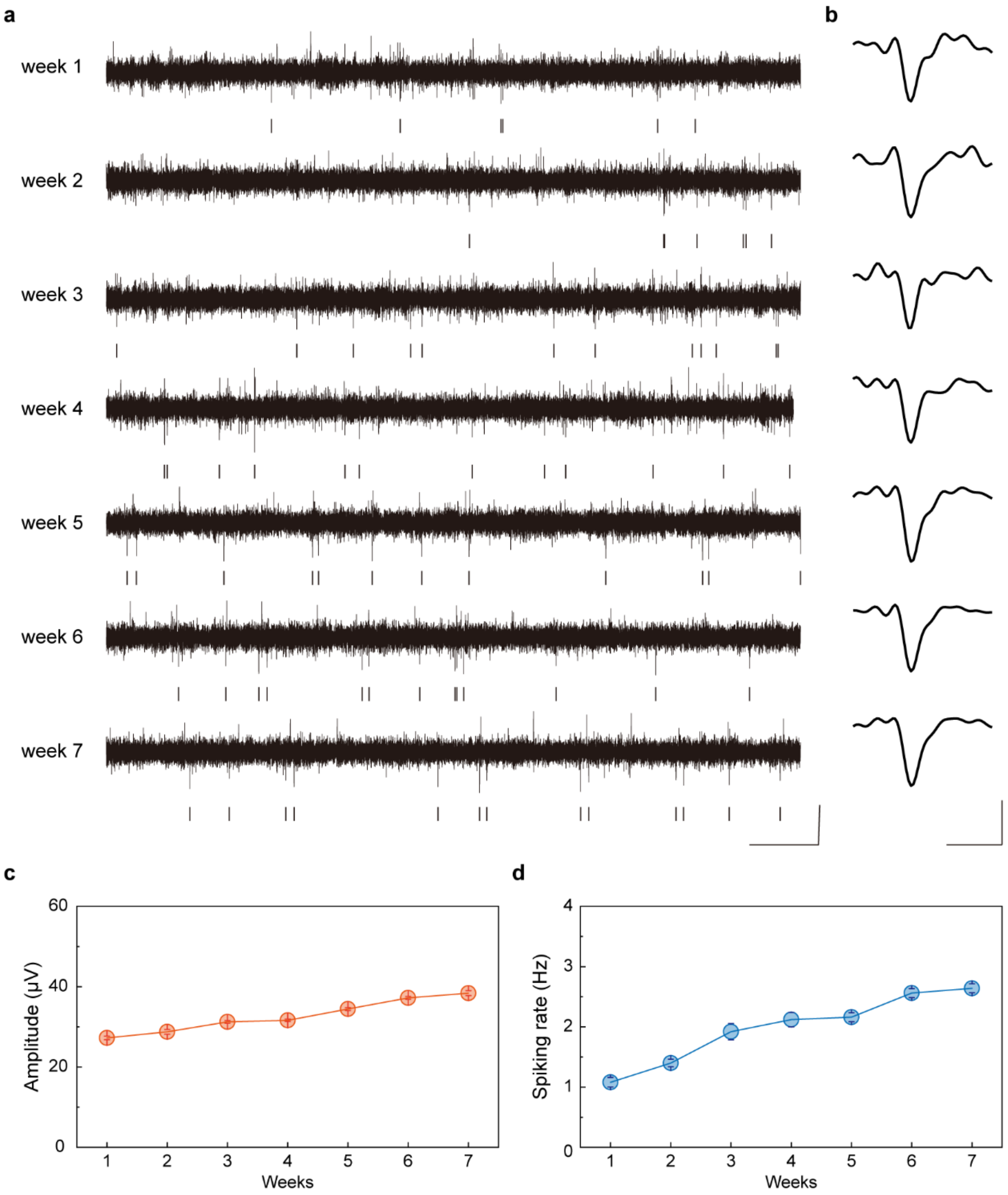

Supplementary Figure 18. Long-term continuous monitoring of neural signals from a single brain organoid for 7 weeks. **a**, Representative single-unit potentials and raster plot of each week. Scale bars, 20 μV (vertical), 0.5 s (horizontal). **b,** Representative spikeforms of each week. Scale bars, 20 μV (vertical), 1 ms (horizontal). **c,d,** Changes in neural signals for 7 weeks (n = 5 electrodes per each week); (**c**) Amplitude of spikes, (**d**) Spiking rates. All data are presented as mean ± s.e.m.

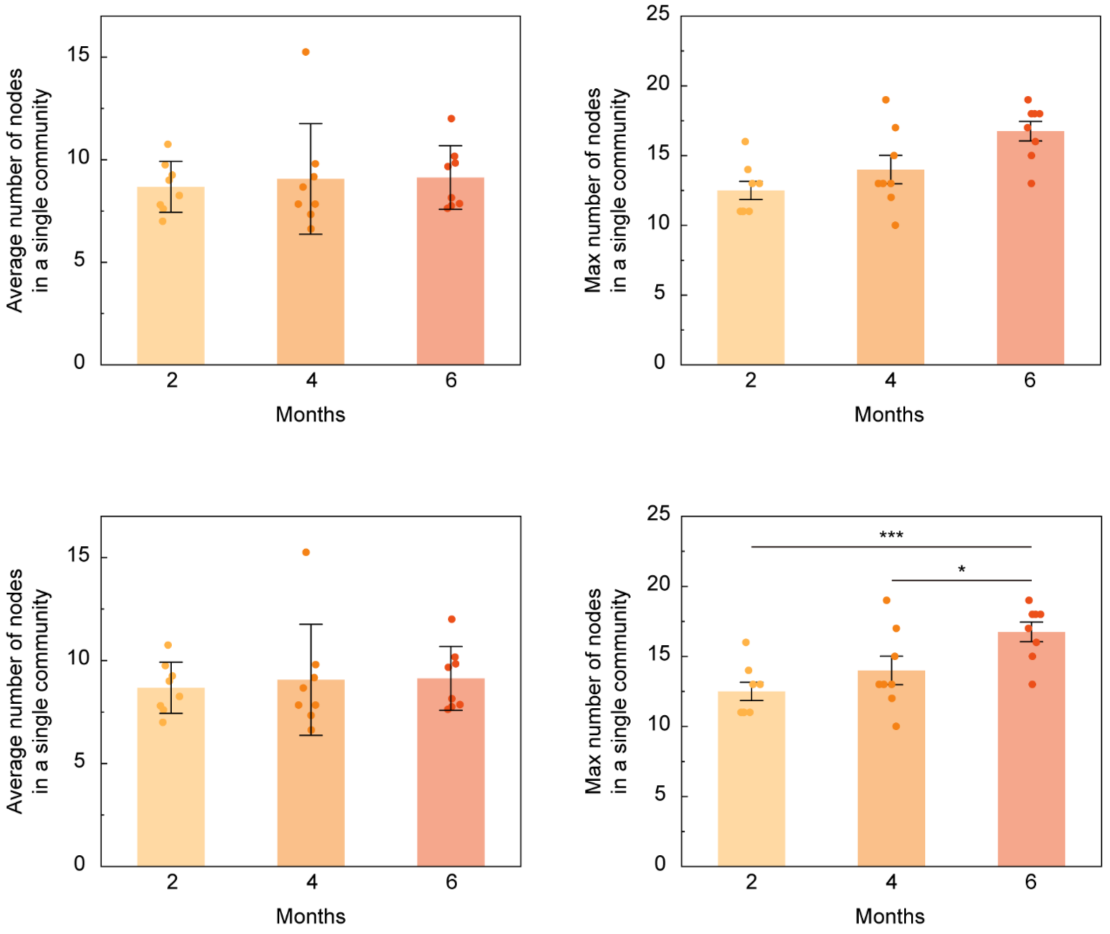

Supplementary Figure 19. Average number of nodes in a single community (left) and maximum number of nodes in a single community (right, p = 0.02149 for 4 and 6 months; p = 0.0002845 for 2 and 6 months) during the maturation-span (n = 8 independent organoids per each month). All data are presented as mean ± s.e.m., Statistical differences were determined with unpaired, one-sided t-test; $*p<0.05$, $***p<0.001$.

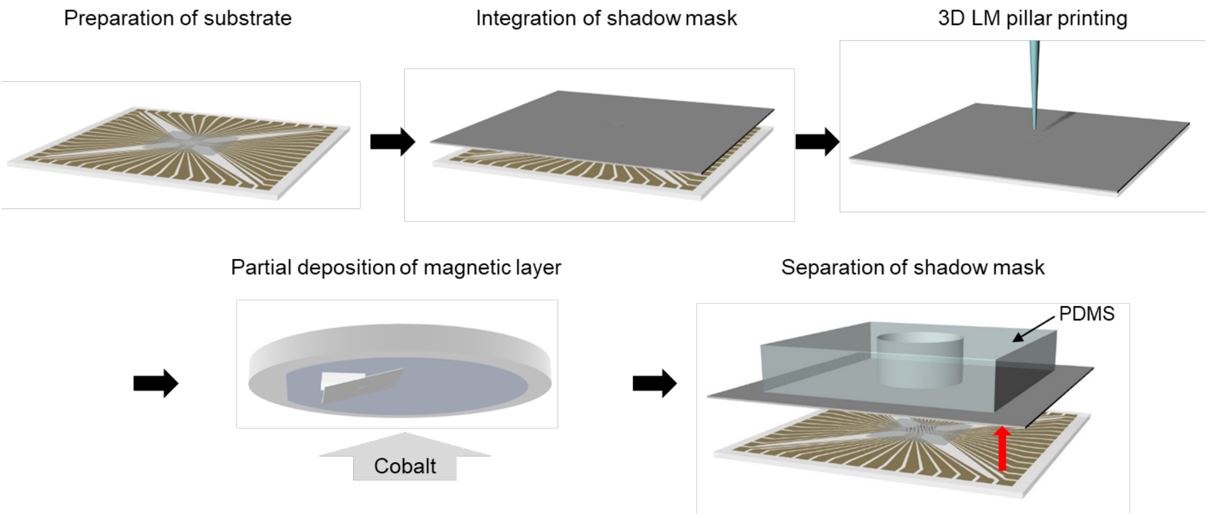

Supplementary Figure 20. Schematic illustration describing the partial deposition of magnetic layer on 3D liquid metal (LM) multi-electrode array (MEA).

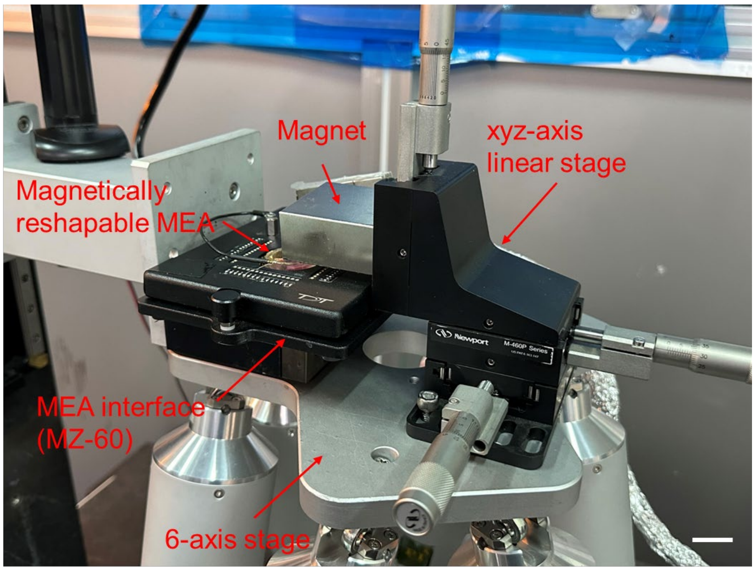

Supplementary Figure 21. The magnetic tilting system with a 6-axis stage enabling the neural recording while tilting the electrodes using the movement of a magnet. Scale bar, 1 cm.

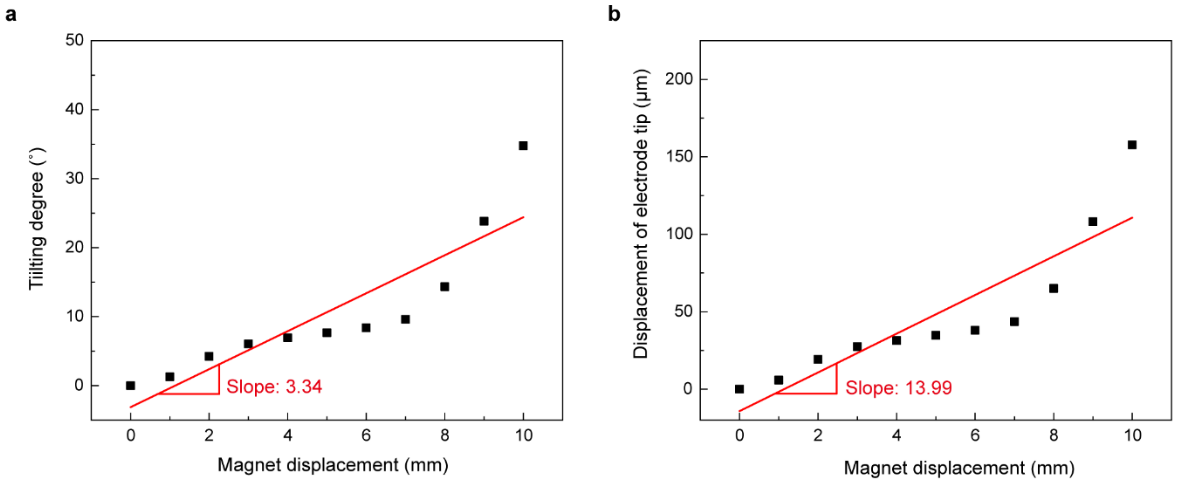

Supplementary Figure 22. Tilting behavior of 3D liquid metal (LM) pillars (height: 260 μm) along the magnet displacement. **a**, Change in tilting degree along the magnet displacement. The slope of linear fit (red line) represents that the magnetically tiltable electrode deflects 3.34 ° for 1 mm of magnet displacement. **b**, Change in displacement of electrode tip along the magnet displacement. The slope of linear fit (red line) represents that the tip of magnetically tiltable electrode shifts 13.99 μm for 1 mm of magnet displacement.

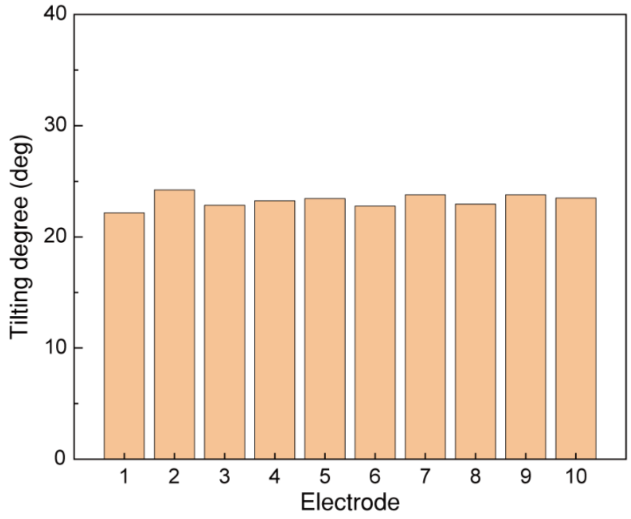

Supplementary Figure 23. Distribution of tilting degrees for 10 electrodes uniformly tilted by a single horizontal movement of a magnet.

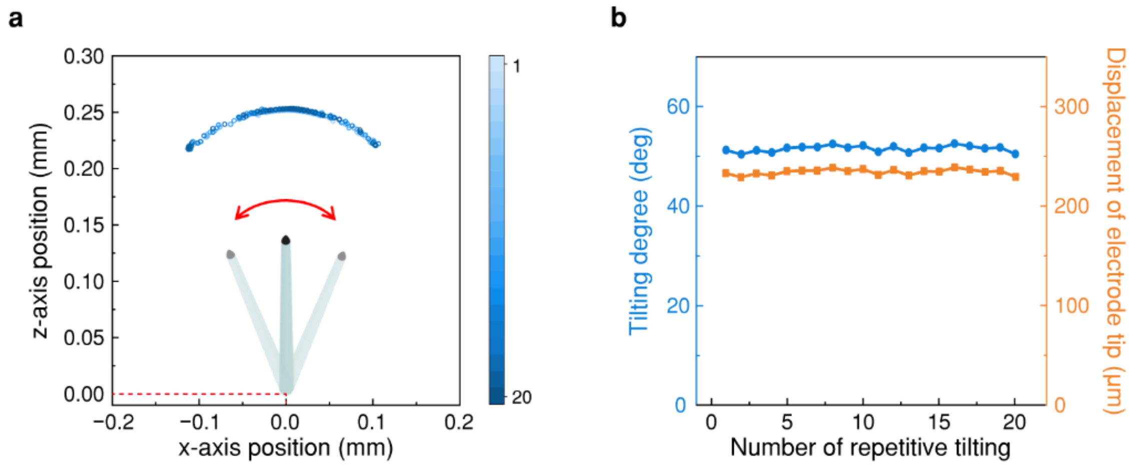

Supplementary Figure 24. Magnetic tilting of an electrode in both directions along the identical trajectory in the xz-plane. The trajectories (**a**), tilting degrees (blue), and displacements (yellow) of the electrode tip (**b**) were observed over 20 cycles of repetitive magnetic tilting. The maximum tilting degree in both directions showed 51.6 ± 0.21°, and the displacement of the electrode tip was 234 μm.

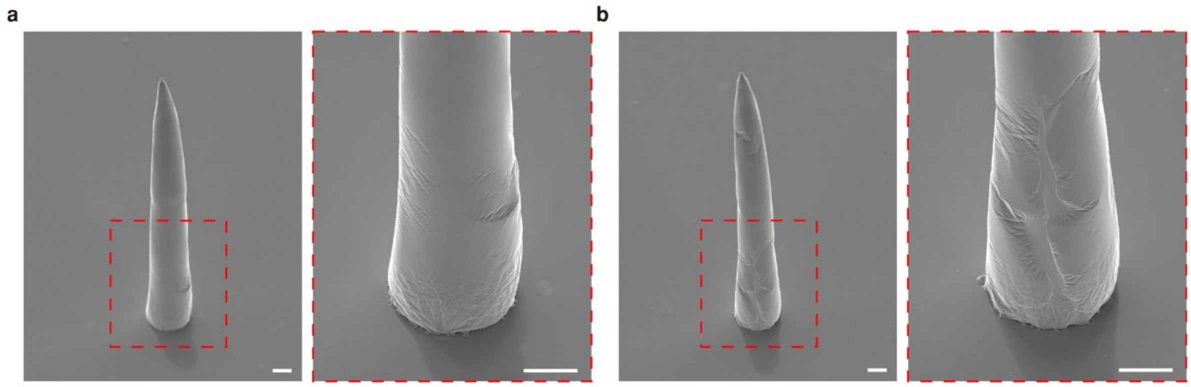

Supplementary Figure 25. Scanning electron microscopy (SEM) images magnetically tiltable electrode; (**a**) Before magnetic tilting, (**b**) After 20 cycles of repetitive magnetic tilting. Scale bars, 10 μm. This experiment was independently repeated more than ten times with similar results.

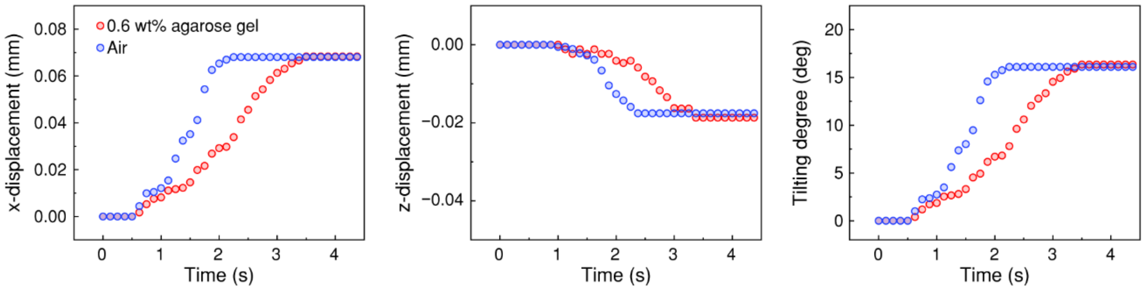

Supplementary Figure 26. Comparison of magnetic tilting of the electrode in air (blue dots) and in the 0.6 wt% agarose gel (red dots) during magnet movement at 3 mm/s; x-displacement (left), z-displacement (middle), and tilting degree (right).

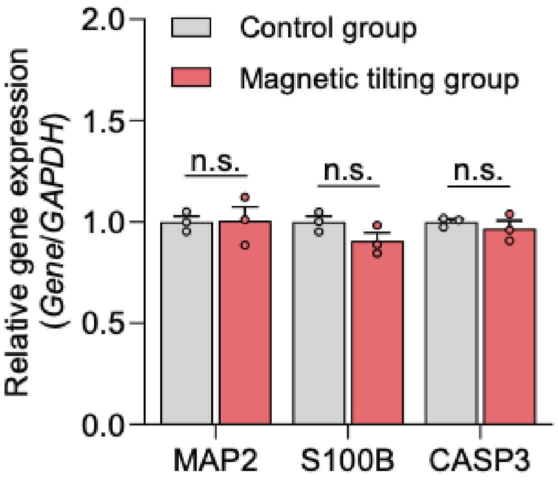

Supplementary Figure 27. The qPCR analysis of cortical organoids in the magnetic tilting group (n = 3, biological replicates), which underwent magnetic tilting (tilting angle: 20˚ for 3 hours), and the control group (n = 3, biological replicates), which was not exposed to the applied magnetic field. All data are presented as mean ± s.e.m. Statistical differences were determined using an unpaired, two-sided t-test. n.s. indicates not significant.

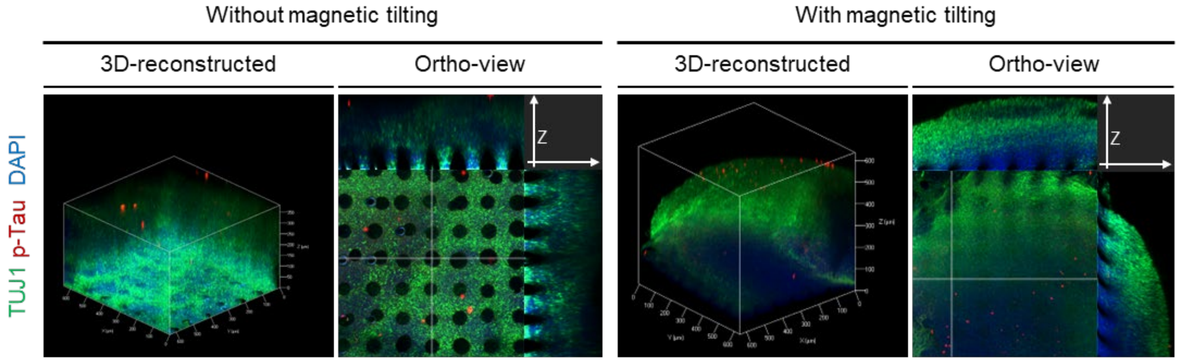

Supplementary Figure 28. Whole-mount 3D imaging of optically cleared organoids without (left) and with (right) magnetic titling of 3D LM electrodes. For the organoid with magnetic tilting, the organoid was fixed with the 3D LM electrodes at the tilted position. The organoids were stained with TUJ1, phosphorylated Tau (p-Tau), and DAPI for investigation of internal architecture and damage of organoids.

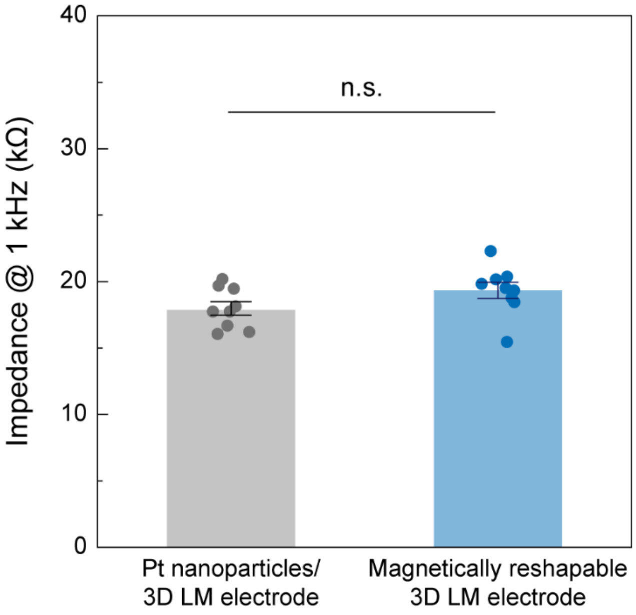

Supplementary Figure 29. Comparison of impedance between magnetically tiltable 3D LM electrodes and pristine 3D LM electrodes (n = 9 electrodes per each group; p = 0.0530). Data are presented as mean ± s.e.m. Statistical differences were determined with unpaired, one-sided t-test; n.s. indicates not significant ($p > 0.05$).

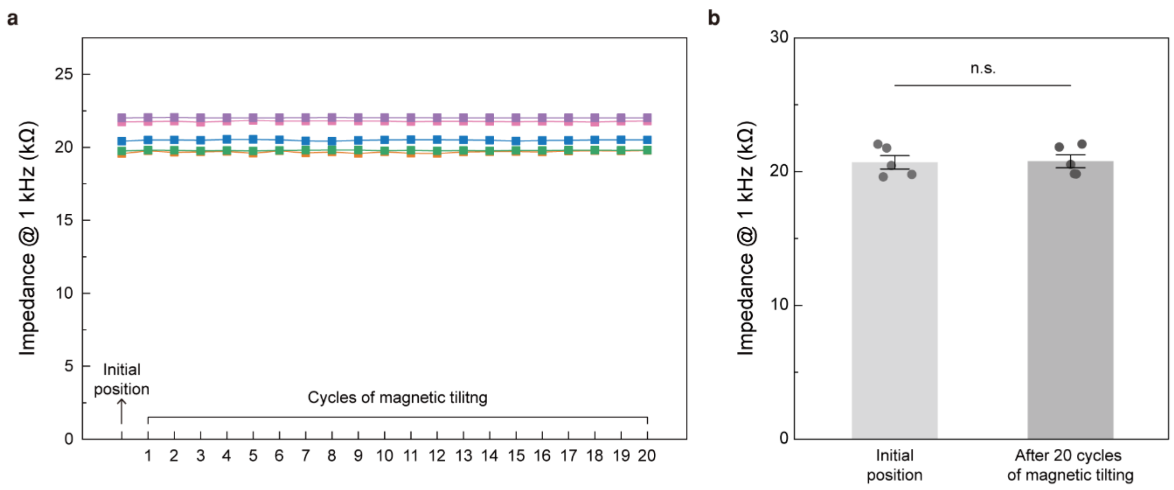

Supplementary Figure 30. Functional stability of magnetically tiltable 3D liquid metal (LM) electrodes. **a,** Changes in impedance over 20 cycles of repetitive magnetic tilting (n = 5 electrodes; each color represents an individual electrode). **b,** The impedance of the electrodes shows negligible change after repetitive magnetic tilting ($p = 0.4534$). All data are presented as mean ± s.e.m., and statistical differences were determined with unpaired, one-sided t-test; n.s. indicates not significant ($p > 0.05$).

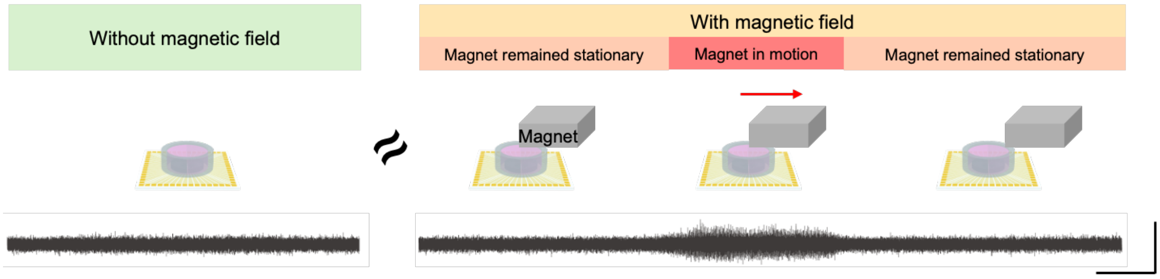

Supplementary Figure 31. The noise level of magnetically tiltable 3D liquid metal (LM) multi-electrode array (MEA) without (left) and with an external magnetic field during the magnet movement (right). Scale bars, 50 µV (vertical), 1 s (horizontal). The noise level only during magnetic movement increased slightly (~ 52 µV) due to changes in the magnetic field. Since our electrophysiological recordings were conducted only when the magnet remained stationary, the noise induced during the magnet movement did not affect these recordings.

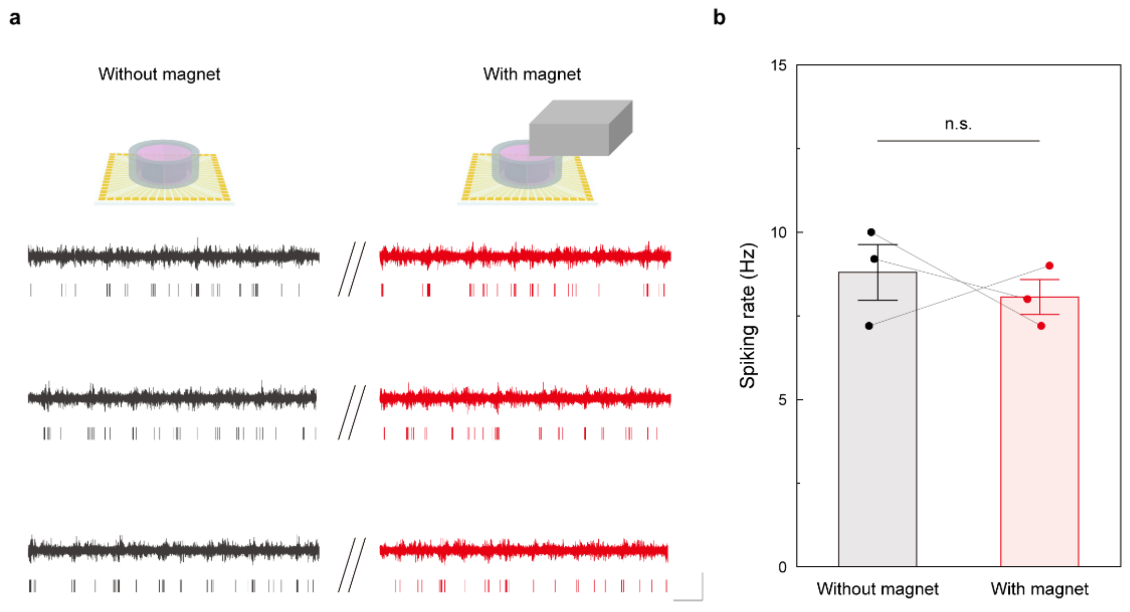

Supplementary Figure 32. Spontaneous neural activities of brain organoids detected by 3D LM MEA without and with external magnetic field. **a**, Single-unit potentials from a single brain organoid recorded by 3D LM MEA without (black) and with (red) external magnetic field (n = 3 electrodes per each group; p = 0.2811). Scale bars, 50 µV (vertical), 0.5 s (horizontal). **b**, Comparison of spiking rates between single-unit potentials recorded without and with external magnetic field. All data are presented as mean ± s.e.m. and statistical differences were determined with unpaired, one-sided t-test; n.s. indicates not significant ($p > 0.05$).

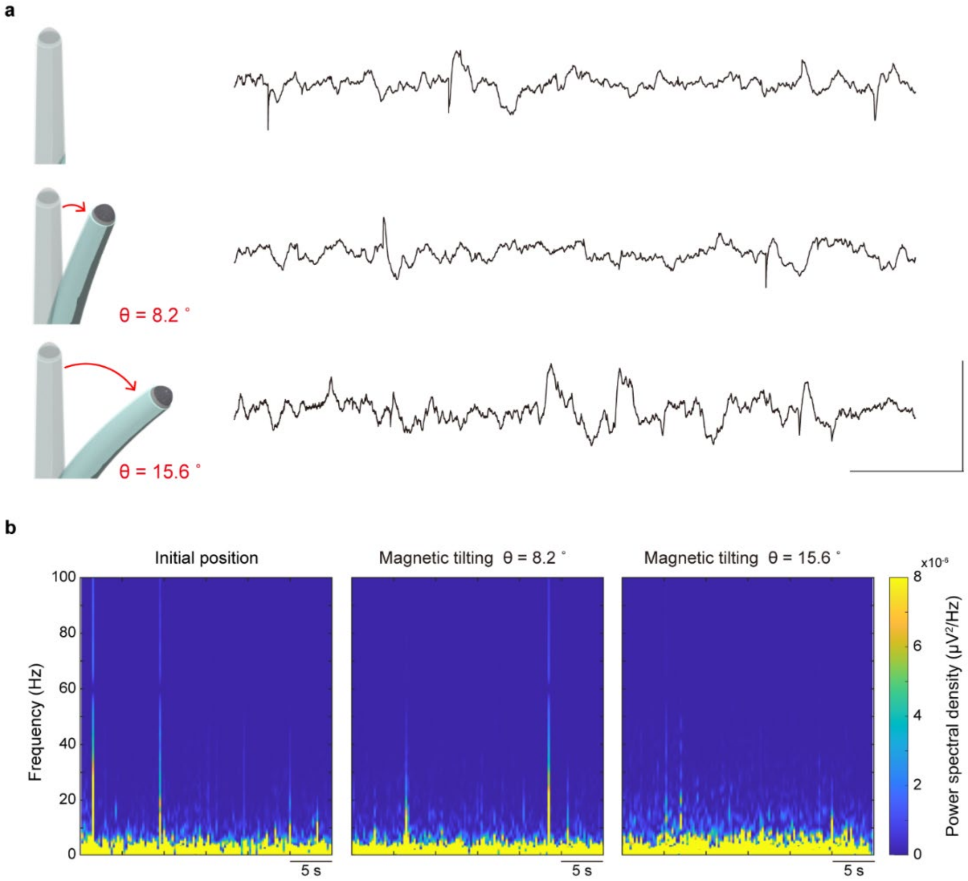

Supplementary Figure 33. a, Local field potential recorded by magnetically reshapable 3D LM MEA. Scale bars, 500 μV (vertical), 5 s (horizontal). **b**, Power spectral density analysis during the magnetic tilting of electrodes.

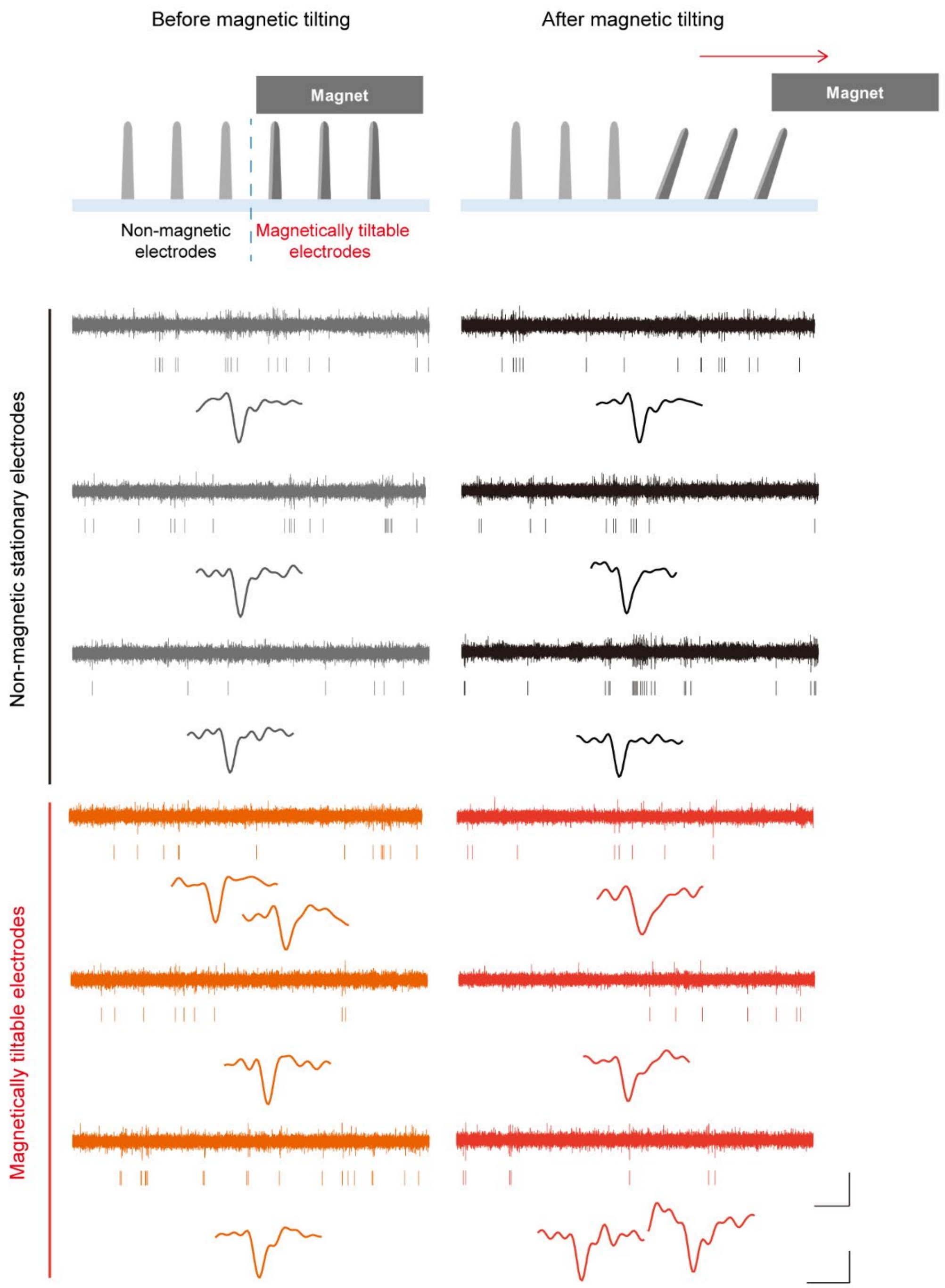

Supplementary Figure 34. Neural activities (single-unit potentials, raster plot, and spikeforms) detected by both non-magnetic stationary electrodes (grey, black) and magnetic electrodes (orange, red) from a single organoid. Each row presents representative single unit potential (top), raster plot (middle), and spikeforms (bottom) from a single electrode. The left column

shows the neural activities before the magnetic tilting and the right column shows them after the magnetic tilting. Scale bars for single-unit potentials, 50 μV (vertical), 0.5 s (horizontal). Scale bars for spikeforms, 20 μV (vertical), 1 ms (horizontal).

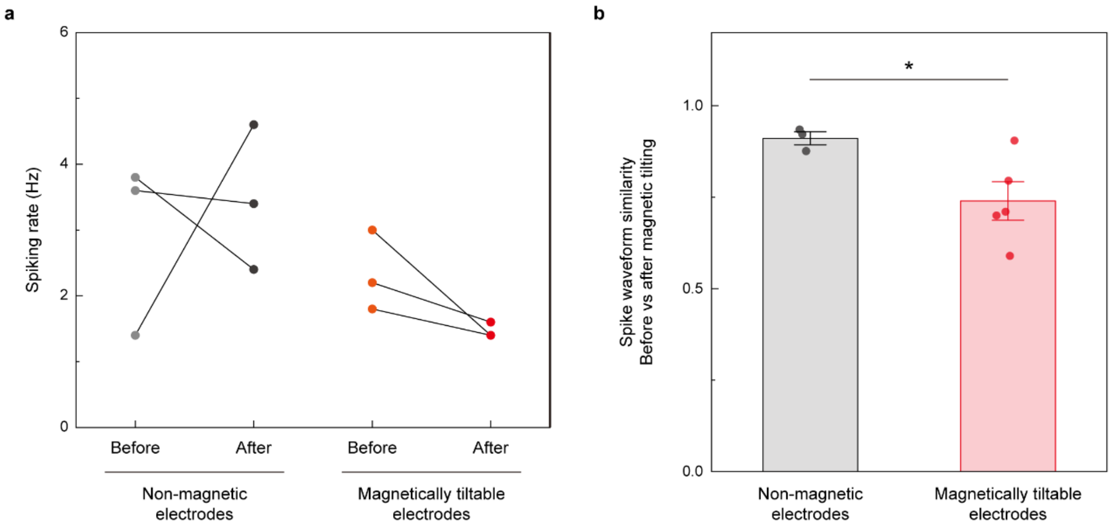

Supplementary Figure 35. Comparison of neural activities between non-magnetic electrodes and magnetically tiltable electrodes. **a**, Changes in spiking rates before and after the magnetic tilting. **b**, Changes in spike waveform similarities between before and after the magnetic tilting for each electrode (p = 0.02668). The similarities of spike waveforms of magnetically tiltable electrodes show lower value than that of non-magnetic stationary electrodes. This result indicates that the magnetically tiltable electrodes effectively shift the recording spots within the organoid to detect the other group of neurons, whereas the non-magnetic stationary electrodes consistently detect the same group of neurons, unaffected by the magnetic tilting. All data are presented as mean ± s.e.m. and statistical differences were determined with unpaired, one-sided t-test; $*p<0.05$.

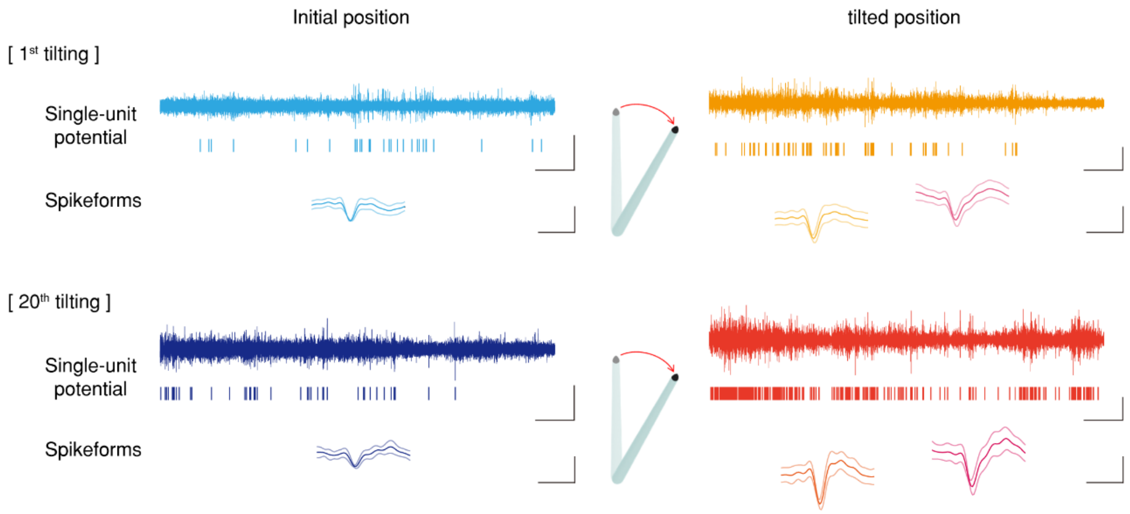

Supplementary Figure 36. Intra-organoid signals at initial (sky blue, navy) and tilted positions (yellow, red) for the 1st and 20th of magnetic tilting cycles. Scale bars for single-unit potential, 100 μV (vertical), 0.5 s (horizontal). Scale bars for spikeforms, 50 μV (vertical), 1 ms (horizontal).

Supplementary Table

References	Type of MEA	Number of electrodes	Surface area of a single electrode	Detectable area of MEA
This work; Magnetically reshapable MEA	3D electrodes array (inserted within the organoid)	60	78.5 μm^2 ↓ 699,496 μm^2 by magnetic tilting	~42,000,000 μm^2
Adv. Mater. 34, 2106829 (2022)	Mesh type (inserted within the organoid)	16	490.625 μm^2 (diameter: 25 μm)	7,850 μm^2
Sci. Adv. 7, abf9153 (2021)	3D interface (surface contact)	25	1962.5 μm^2 (diameter: 50 μm)	49,062.5 μm^2
Nat. Biotechnol. Online published (2024)	Kirigami electronics (surface contact)	32	490.625 μm^2 (diameter: 25 μm)	15,700 μm^2

Supplementary Table 1. Comparison of the detectable area of our magnetically reshapable 3D liquid metal (LM) multi-electrode array (MEA) to other MEA systems in previous studies.

Supplementary Notes

Supplementary Note 1 | Direct printing process of interconnects and 3D LM pillars

First, the stage was adjusted in the z-axis for a substrate to reach a meniscus of EGaIn formed at the end tip of nozzle. Second, the stage controller was programmed to move for printing interconnects. Then, when the nozzle was located at the point where the 3D pillar should be printed, the stage made circles in the xy plane for adhesion of EGaIn on the substrate. Then, the stage moved down along the z-axis until reaching a targeting pillar height. Lastly, a swift descent of stage with a velocity of 10 times higher than printing velocity disconnected a pillar-shaped EGaIn from the nozzle (Supplementary Movie 1). As the printed EGaIn patterns formed an ultrathin oxide solid shell (thickness: ~ 1 nm) upon exposure to the air, the 3D pillar structure was maintained while the liquid phase was confined in the solid oxide shell. Thus, the velocity of stage movement, referred to as printing velocity, and applied pressure on the nozzle should be controlled to print 3D LM pillars with exact target heights. The printing velocity needed to match a speed of instantaneous formation of the oxide shell. Also, since the applied pressure determined a volume of EGaIn which needed to be confined within this ultrathin oxide shell, this parameter needed to be carefully controlled.

Supplementary Note 2 | Calculation of synchronization score

First, two electrodes out of the 60 electrodes in our MEA were selected to be analyzed. The position of spikes along time traces was compared between electrodes, and the temporal difference between spikes was calculated. The degree of proximity between spikes on one electrode and spikes on the other electrode at a time point was reflected in a synchronization score ranging from 0 to 1. Highly synchronized electrodes exhibited synchronization scores close to 1.

Supplementary Note 3 | Computational method for neural community

To determine the neural communities throughout the organoid, each electrode was defined as a node, and two nodes were connected with a line if the synchronization score exceeded 0.5. The degree of complexity of the line between nodes was reflected in the modularity. The Louvain algorithm identified neural community by repeating random node groupings until it found the group with the highest modularity. An arbitrary group of nodes that exhibited the highest modularity and most complex circuitry was designated as a single neural community. These series of computations were conducted through custom codes in MATLAB and Python.

Supplementary Note 4 | Precise controllability of our magnetic tilting system

To investigate the precise controllability of our magnetic tilting system, we calculate the minimum tilting degree and the minimum displacement of electrode tip when the magnet moves 0.5 μm, which is the minimum magnet displacement under our magnetic tilting system. Since the bottom of the magnetically tiltable electrodes are fixed on the substrate, the tilting degree of the electrode tip depends on the torque force exerted by the magnetic field, as described by the equation (1),

$$\theta = \frac{\tau \cdot L}{E \cdot I} \qquad (1)$$

where θ is the tilting degree of magnetically tiltable electrode, τ is the torque force exerted on the electrode by the magnetic field gradient, L is the height of the electrode, E is the elastic modulus of the electrode, and I is the area moment of inertia. As the magnetically tiltable electrodes are forced to be tilted by the magnetic layer on the surface under the magnetic field gradient, τ is described by the following equation (2),

$$\tau = L \cdot F \cdot N_{Co} = L \cdot m_{Co} \nabla B \cdot N_{Co} \approx L \cdot m_{Co} \cdot \frac{3B}{r} \cdot N_{Co} \qquad (2)$$

where F is the exerted force on the electrode, N_{Co} is the number of cobalt atom of the electrode, m_{Co} is the magnetic moment of cobalt, which is $1.72\mu_B$ (Bohr magneton; $\mu_B = 9.274 \times 10^{-24}\ A \cdot m^2$), ∇B is the magnetic field gradient, B is the magnetic flux density, and r is the displacement of the magnet. The magnet of our magnetic tilting system exhibits the magnetic flux density of 285 mT, and the magnetic flux density at the distance of 2 cm from the magnet is 4.45 mT since the magnetic field decreases by the 3 orders of magnitude. Furthermore, considering the magnetically tiltable electrode (diameter: 20 μm, height: 260 μm) is covered by 120 nm thickness of cobalt (density: 8.9 g/cm^2) on the half side, N_{Co} is $8.913 \times$

10^{13} atom . As a result, τ is calculated as 9.872×10^{-9} Nm.

As the magnetically tiltable electrodes are composed of EGaIn and cobalt, the elastic modulus of the electrodes is calculated considering the volume percentages of each material, which is 98.8% and 1.2%, respectively. Considering the elastic modulus of each material (210 GPa for cobalt and 210 kPa for EGaIn), the elastic modulus of the magnetically tiltable electrode is 2.53 GPa. Consequently, the minimum tilting degree of the magnetically tiltable electrode is calculated as 0.002 ° and the minimum displacement of electrode tip is 0.009 μm.

Supplementary Note 5 | Magnetic tilting of 3D LM electrodes in viscous medium

When the electrode is tilted in air, the air viscosity negligibly affects the tilting performance. In this case, the force exerted on the electrodes, excluding gravity, is the magnetic force, described by the equation (3),

$$F_{mag} = \mu \cdot V \cdot \nabla B \quad (3)$$

where F_{mag} is the magnetic force exerted on the electrode, μ is the magnetic permeability of the ferromagnetic material, V is the volume of the electrode, and ∇B is the gradient of the magnetic field. When the magnetically tiltable electrode is embedded within the brain organoid, the viscosity induces drag force on the electrode, as described by the equation (4),

$$F_{drag} = 6\pi\eta r v \quad (4)$$

where F_{drag} is the drag force exerted on the electrode due to the viscosity, η is the viscosity of the medium, r, and v are the radius and velocity of the electrode, respectively. From these equations, the tilting velocity of the electrode can be described by the following equation (5),

$$v = \frac{\mu V \nabla B}{6\pi\eta r} \quad (5)$$

which shows the inversely proportional relationship between the viscosity and the tilting speed. However, when sufficient time is provided for the electrode to tilt, the magnetic force induced on the electrode is strong enough to overcome the drag force, resulting the identical position of the electrode regardless of the viscosity of the medium (although the tilting speed is different). This corresponds to the results shown in Supplementary Fig. 26.
