# Supplementary material for: Magnetically reshapable 3D multi-electrode arrays of liquid metals for electrophysiological analysis of brain organoids
Source: Nat Commun. 2025 Feb 27;16:2011. doi: 10.1038/s41467-024-55752-3 (PMC11868496; doi:10.1038/s41467-024-55752-3)
Supplement: Supplementary file 2 — Description of Additional Supplementary Files [file 41467_2024_55752_MOESM2_ESM.pdf]

Description of Additional Supplementary Files

Supplementary Video 1. Direct printing of LM interconnects and 3D pillars.

Supplementary Video 2. Organoid insertion process.

Supplementary Video 3. Magnetic tilting system for simultaneous neural recording.

Supplementary Video 4. Magnetic tilting of 3D LM MEA.

Supplementary Video 5. Repetitive magnetic tilting of 3D LM electrode.

Supplementary Video 6. Magnetic tilting in both directions along the identical trajectory in xzplane.

Supplementary Video 7. Magnetic tilting of electrodes from multi-perspectives.

Supplementary Video 8. Magnetic tilting of a 3D LM electrode in air and in a 0.6 wt% agarose gel.

Supplementary Video 9. Immobilization of brain organoid under magnetic tilting of electrode
