# Supplementary material for: Magnetically reshapable 3D multi-electrode arrays of liquid metals for electrophysiological analysis of brain organoids
Source: Nat Commun. 2025 Feb 27;16:2011. doi: 10.1038/s41467-024-55752-3 (PMC11868496; doi:10.1038/s41467-024-55752-3)
Supplement: Supplementary file 12 — Reporting Summary [file 41467_2024_55752_MOESM12_ESM.pdf]

nature portfolio

Corresponding author(s): Jang-Ung Park, Seung-Woo Cho, Jae-Hyun Lee

Last updated by author(s): Dec 20, 2024

Reporting Summary

Nature Portfolio wishes to improve the reproducibility of the work that we publish. This form provides structure for consistency and transparency in reporting. For further information on Nature Portfolio policies, see our Editorial Policies and the Editorial Policy Checklist.

Statistics

For all statistical analyses, confirm that the following items are present in the figure legend, table legend, main text, or Methods section.

n/a	Confirmed	
☐	☒	The exact sample size (*n*) for each experimental group/condition, given as a discrete number and unit of measurement
☐	☒	A statement on whether measurements were taken from distinct samples or whether the same sample was measured repeatedly
☐	☒	The statistical test(s) used AND whether they are one- or two-sided<br>*Only common tests should be described solely by name; describe more complex techniques in the Methods section.*
☒	☐	A description of all covariates tested
☒	☐	A description of any assumptions or corrections, such as tests of normality and adjustment for multiple comparisons
☐	☒	A full description of the statistical parameters including central tendency (e.g. means) or other basic estimates (e.g. regression coefficient) AND variation (e.g. standard deviation) or associated estimates of uncertainty (e.g. confidence intervals)
☐	☒	For null hypothesis testing, the test statistic (e.g. *F*, *t*, *r*) with confidence intervals, effect sizes, degrees of freedom and *P* value noted<br>*Give P values as exact values whenever suitable.*
☒	☐	For Bayesian analysis, information on the choice of priors and Markov chain Monte Carlo settings
☒	☐	For hierarchical and complex designs, identification of the appropriate level for tests and full reporting of outcomes
☒	☐	Estimates of effect sizes (e.g. Cohen's *d*, Pearson's *r*), indicating how they were calculated

Our web collection on statistics for biologists contains articles on many of the points above.

Software and code

Policy information about availability of computer code

Data collection	Synapse (Tucker-Davis Technologies, Inc.)<br>VersaStudio
Data analysis	Synapse (Tucker-Davis Technologies, Inc.)<br>Origin 2024<br>ImageJ 1.53t<br>Matlab R2022b<br>Python (3.9.2)<br>Other analyses were done with custom Matlab code. The source code is available under the license of CC0 at Code Ocean (https://doi.org/10.24433/CO.7867348.v1).

For manuscripts utilizing custom algorithms or software that are central to the research but not yet described in published literature, software must be made available to editors and reviewers. We strongly encourage code deposition in a community repository (e.g. GitHub). See the Nature Portfolio guidelines for submitting code & software for further information.

Data

Policy information about availability of data

All manuscripts must include a data availability statement. This statement should provide the following information, where applicable:

- Accession codes, unique identifiers, or web links for publicly available datasets
- A description of any restrictions on data availability
- For clinical datasets or third party data, please ensure that the statement adheres to our policy

The main data supporting the results in this study are available within the paper and its Supplementary Information. The datasets generated during the study are too large to be publicly shared. Source data are available at Figshare (https://doi.org/10.6084/m9.figshare.24764508). Source data are provided with this paper for reproducing all Figures in the manuscript and Supplementary Information.

Research involving human participants, their data, or biological material

Policy information about studies with human participants or human data. See also policy information about sex, gender (identity/presentation), and sexual orientation and race, ethnicity and racism.

Reporting on sex and gender	The study did not involve human research participants.
Reporting on race, ethnicity, or other socially relevant groupings	The study did not involve human research participants.
Population characteristics	The study did not involve human research participants.
Recruitment	The study did not involve human research participants.
Ethics oversight	The study did not involve human research participants.

Note that full information on the approval of the study protocol must also be provided in the manuscript.

Field-specific reporting

Please select the one below that is the best fit for your research. If you are not sure, read the appropriate sections before making your selection.

☒ Life sciences ☐ Behavioural & social sciences ☐ Ecological, evolutionary & environmental sciences

For a reference copy of the document with all sections, see nature.com/documents/nr-reporting-summary-flat.pdf

Life sciences study design

All studies must disclose on these points even when the disclosure is negative.

Sample size	Sample sizes were determined by the number of biological and technical replicates necessary to observe the effect was real.
Data exclusions	There were no exclusions.
Replication	At least three organoids were performed in each case of this study, and all experimental findings were reliably reproduced at least three times.
Randomization	All samples were allocated randomly into experimental groups.
Blinding	Blinding was not relevant, because a blinding process wouldn't influence the sampling result.

Reporting for specific materials, systems and methods

We require information from authors about some types of materials, experimental systems and methods used in many studies. Here, indicate whether each material, system or method listed is relevant to your study. If you are not sure if a list item applies to your research, read the appropriate section before selecting a response.

Materials & experimental systems

n/a	Involved in the study
	☒ Antibodies
	☒ Eukaryotic cell lines
☒	Palaeontology and archaeology
☒	Animals and other organisms
☒	Clinical data
☒	Dual use research of concern
☒	Plants

Methods

n/a	Involved in the study
☒	ChIP-seq
☒	Flow cytometry
☒	MRI-based neuroimaging

Antibodies

Antibodies used

Antibody name (supplier; catalog number): Mouse Anti-TUJ1 (BioLegend; 801202), Rabbit Anti-TUJ1 (Cell Signaling Technology; 5568S), Rabbit Anti-SOX2 (Millipore; AB5603), Rabbit Anti-MAP2 (Cell Signaling Technology; 4542S), Mouse Anti-GFAP (Millipore; MAB3402), Rabbit Anti-VGLUT1 (Synaptic Systems; 135 302), Rat Anti-Ctip2 (Abcam; ab18465), Mouse Anti-Human SATB2 (Abcam; ab51502), Mouse Anti-Nestin (Millipore; MAB5326), Mouse Anti-Phospho-Tau (Thermo Fisher Scientific; MN1020),
Alexa Fluor 488 goat anti-mouse IgG (Thermo Fisher; A11001), Alexa Fluor 488 goat anti-rabbit IgG (Thermo Fisher; A11008), Alexa Fluor 488 goat anti-rat IgG (Thermo Fisher; A11006), Alexa Fluor 594 goat anti-mouse IgG (Thermo Fisher; A11005), Alexa Fluor 594 goat anti-rabbit IgG (Thermo Fisher; A11012), Alexa Flour 555 goat anti-mouse IgG1 (Thermo Fisher; A21127), Alexa Flour 488 goat anti-Mouse IgG2a (Thermo Fisher; A21131)

Validation

All antibodies listed above are commercially available and have been verified by many references provided on the website of the companies that sell antibodies (links below).

Mouse Anti-TUJ1 (BioLegend; 801202)
Validation Refs. from the manufacturer's datasheet: https://www.biolegend.com/fr-lu/explore-new-products/purified-anti-tubulin-beta-3-tubb3-antibody-11580?pdf=true&displayInline=true&leftRightMargin=15&topBottomMargin=15&filename=Purified%20anti-Tubulin%20%CE%B2%203%20(TUBB3)%20Antibody.pdf&v=20240814063131

Rabbit Anti-TUJ1 (Cell Signaling Technology; 5568S)
Validation Refs. from the manufacturer's datasheet: https://www.cellsignal.com/products/5568/datasheet?images=1&srsltid=AfmBOorgFXlgrO0yWfNKZNOYn2Qr8dxEki77brb9D0mPJrspXsOT5H0P

Rabbit Anti-SOX2 (Millipore; AB5603)
Validation Refs. from the manufacturer's datasheet: https://www.sigmaaldrich.com/KR/ko/product/mm/ab5603?srsltid=AfmBOorKAaotQpgxi9b6iv7x_WbbqJNCcxVYkI7lmOAeQCJs-ZaLG1WJ

Rabbit Anti-MAP2 (Cell Signaling Technology; 4542S)
Validation Refs. from the manufacturer's datasheet: https://www.cellsignal.com/datasheet.jsp?productId=4542&images=1&srsltid=AfmBOookdIcV-B_ycSr_JCCfZMZN77MLjrBwkPnk7kgR-zFnEqRqcY0r

Mouse Anti-GFAP (Millipore; MAB3402)
Validation Refs. from the manufacturer's datasheet:
https://www.merckmillipore.com/KR/ko/product/Anti-Glial-Fibrillary-Acidic-Protein-Antibody-clone-GA5,MM_NF-MAB3402

Rabbit Anti-VGLUT1 (Synaptic Systems; 135 302)
Validation Refs. from the manufacturer's datasheet:
https://sysy.com/product/135302

Rat Anti-Ctip2 (Abcam; ab18465)
Validation Refs. from the manufacturer's datasheet:
https://www.abcam.com/en-us/products/primary-antibodies/ctip2-antibody-25b6-ab18465#tab=datasheet

Mouse Anti-Human SATB2 (Abcam; ab51502)
Validation Refs. from the manufacturer's datasheet:
https://www.abcam.com/en-us/products/primary-antibodies/satb1-satb2-antibody-satba4b10-c-terminal-ab51502

Mouse Anti-Nestin (Millipore; MAB5326)
Validation Refs. from the manufacturer's datasheet:
https://www.merckmillipore.com/KR/ko/product/Anti-Nestin-Antibody-clone-10C2,MM_NF-MAB5326

Mouse Anti-Phospho-Tau (Thermo Fisher Scientific; MN1020)
Validation Refs. from the manufacturer's datasheet:
https://www.thermofisher.com/antibody/product/Phospho-Tau-Ser202-Thr205-Antibody-clone-AT8-Monoclonal/MN1020

Alexa Fluor 488 goat anti-mouse IgG (Thermo Fisher; A11001)
Validation Refs. from the manufacturer's datasheet: https://www.thermofisher.com/order/genome-database/dataSheetPdf?producttype=antibody&productsubtype=antibody_secondary&productId=A-11001&version=Local

Alexa Fluor 488 goat anti-rabbit IgG (ThermoFisher; A11008)

Validation Refs. from the manufacturer's datasheet: https://www.thermofisher.com/order/genome-database/dataSheetPdf?producttype=antibody&productsubtype=antibody_secondary&productId=A-11008&version=Local

Alexa Fluor 488 goat anti-rat IgG (Thermo Fisher; A11006)
Validation Refs. from the manufacturer's datasheet: https://www.thermofisher.com/order/genome-database/dataSheetPdf?producttype=antibody&productsubtype=antibody_secondary&productId=A-11006&version=Local

Alexa Fluor 594 goat anti-mouse IgG (Thermo Fisher; A11005)
Validation Refs. from the manufacturer's datasheet:
https://www.thermofisher.com/antibody/product/Goat-anti-Mouse-IgG-H-L-Cross-Adsorbed-Secondary-Antibody-Polyclonal/A-11005

Alexa Fluor 594 goat anti-rabbit IgG (Thermo Fisher; A11012)
Validation Refs. from the manufacturer's datasheet:
https://www.thermofisher.com/antibody/product/Goat-anti-Rabbit-IgG-H-L-Cross-Adsorbed-Secondary-Antibody-Polyclonal/A-11012

Alexa Flour 555 goat anti-mouse IgG1 (Thermo Fisher; A21127)
Validation Refs. from the manufacturer's datasheet:
https://www.thermofisher.com/antibody/product/Goat-anti-Mouse-IgG1-Cross-Adsorbed-Secondary-Antibody-Polyclonal/A-21127

Alexa Flour 488 goat anti-Mouse IgG2a (Thermo Fisher; A21131)
Validation Refs. from the manufacturer's datasheet:
https://www.thermofisher.com/antibody/product/Goat-anti-Mouse-IgG2a-Cross-Adsorbed-Secondary-Antibody-Polyclonal/A-21131

Eukaryotic cell lines

Policy information about cell lines and Sex and Gender in Research

Cell line source(s)	The human induced pluripotent stem cells (hiPSCs; Female; KYOU-DXR0109B) were acquired from American Type Culture Collection.
Authentication	The use of human iPSCs was approved by the Institutional Review Board (IRB) of Yonsei University (Permit Number: 7001988-202309-BR-1066-02E).
Mycoplasma contamination	The hiPSCs were regularly checked for mycoplasma contamination.
Commonly misidentified lines (See ICLAC register)	No commonly misidentified lines were used.

Plants

Seed stocks	The study did not involve plant samples.
Novel plant genotypes	The study did not involve plant samples.
Authentication	The study did not involve plant samples.
